# Supplementary material for: A fault-intrusion-tolerant system and deadline-aware algorithm for scheduling scientific workflow in the cloud
Source: PeerJ Comput Sci. 2021 Nov 2;7:e747. doi: 10.7717/peerj-cs.747 (PMC8576554; doi:10.7717/peerj-cs.747)
Supplement: Supplemental Information 6 [file peerj-cs-07-747-s006.zip › Myproject1.1/docs/org/workflowsim/clustering/package-use.html]

Uses of Package org.workflowsim.clustering


JavaScript is disabled on your browser.


- Overview
- Package
- Class
- Use
- Tree
- Deprecated
- Index
- Help

- Prev
- Next

- Frames
- No Frames

- All Classes

# Uses of Package org.workflowsim.clustering

- Packages that use org.workflowsim.clustering

  | Package | Description |
  | --- | --- |
  |  |  |
  | --- | --- |
  | org.workflowsim |  |
  | org.workflowsim.clustering |  |
  | org.workflowsim.clustering.balancing |  |
  | org.workflowsim.clustering.balancing.methods |  |
  | org.workflowsim.clustering.balancing.metrics |  |
- Classes in org.workflowsim.clustering used by org.workflowsim

  Class and Description

  BasicClustering

  The default clustering does no clustering at all, just map a task to a job
- Classes in org.workflowsim.clustering used by org.workflowsim.clustering

  Class and Description

  BasicClustering

  The default clustering does no clustering at all, just map a task to a job

  ClusteringInterface

  The ClusteringInterface for all clustering methods

  TaskSet

  TaskSet is a group of tasks used only in clustering
- Classes in org.workflowsim.clustering used by org.workflowsim.clustering.balancing

  Class and Description

  BasicClustering

  The default clustering does no clustering at all, just map a task to a job

  ClusteringInterface

  The ClusteringInterface for all clustering methods

  TaskSet

  TaskSet is a group of tasks used only in clustering
- Classes in org.workflowsim.clustering used by org.workflowsim.clustering.balancing.methods

  Class and Description

  TaskSet

  TaskSet is a group of tasks used only in clustering
- Classes in org.workflowsim.clustering used by org.workflowsim.clustering.balancing.metrics

  Class and Description

  TaskSet

  TaskSet is a group of tasks used only in clustering

- Overview
- Package
- Class
- Use
- Tree
- Deprecated
- Index
- Help

- Prev
- Next

- Frames
- No Frames

- All Classes
